# The impact of research intercalation during medical school on post-graduate career progression

**DOI:** 10.1186/s12909-020-02478-7

**Published:** 2021-01-08

**Authors:** Antony K. Sorial, Morgan Harrison-Holland, Helen S. Young

**Affiliations:** 1 Biosciences Institute, Newcastle University, Newcastle Upon Tyne, UK; 2 Division of Musculoskeletal and Dermatological Sciences, The University of Manchester, Oxford Road, Manchester, UK

## Abstract

**Background:**

Medical students at The University of Manchester have the option of research intercalation on the Master of Research programme. There is a paucity of evidence for the benefits of research intercalation. However, we hypothesised that research intercalation would accelerate post-graduate career progression and aimed to objectively measure the career enhancing impact, quantify the benefits and determine the alumni perception of research intercalation.

**Methods:**

Data was collected retrospectively by electronic questionnaire (in 2018) from those commencing research intercalation between 2005 and 2012.

**Results:**

Participants (*n*=52) returned questionnaires (68% response), demonstrating that the cohort had completed 67 postgraduate qualifications, published 304 manuscripts (median 3 publications per person (PP); range: 0–53) and made 430 presentations (median 7 PP; range: 0–37). Alumni had been awarded 49 research grants; funding disclosed on 43% totalled £823,000. Career progression of 73% of alumni had taken the minimum number of years; 27% took longer due to time spent working abroad or to gain additional experience prior to specialty training. Fifty-five publications and 71 presentations were directly related to MRes projects.

**Conclusion:**

Research intercalation provides graduates with an opportunity to learn valuable transferrable skills, contribute to translational research, and objectively enhances medical career progression.

**Supplementary Information:**

The online version contains supplementary material available at 10.1186/s12909-020-02478-7.

## Background

Intercalation involves taking time out of the medicine undergraduate programme in order to complete a separate but (usually) related degree [1]. It is widely encouraged at many Medical Schools and incorporated into the undergraduate curricula in some institutions [1]. Over the last several years the range of intercalation opportunities has risen significantly with degree options ranging from Bachelor, Masters and Doctorate level study across a widening range of subject areas including global health and the humanities [1].

The National Health Service (NHS) Plan, published in 2000, made a commitment to i) increase the number of consultants in the NHS and to ii) modernise the Senior House Officer grade [2]. The strategy of Modernising Medical Careers (MMC) was introduced in 2003 as the proposed mechanism to achieve both these targets [2]. The focus of MMC was to improve patient care through the provision of a transparent and efficient career path for doctors (8–10 years for specialty training and 5 years for general practice) supported by high quality medical education [3]. However, MMC in combination with the restrictions of the European Working Time Directive, resulted in a real-time reduction in the length of training required to reach Consultant grade from an average of 21,000 to 6000 h [4]. With junior doctors required to make a choice of specialty at an earlier stage in their careers than previously, under-graduate training and opportunities became increasingly important. In 2005, the academic careers sub-committee of MMC and the UK Clinical Research Collaboration published the Walport report [5]. Walport highlighted three major barriers to clinical academic careers: lack of a transparent career structure, lack of flexibility in training and a shortage of supported posts when training is completed. The Walport report offered solutions to each of these issues and established the clinical academic career pathway with, built-in flexibility, which permitted individuals to move between clinical and academic component [5, 6].

There is a relative paucity of objective evidence for the benefits of intercalation although medical students are aware of its potential drawbacks including additional year(s) of study in an already extended programme (compared to their non-medical peers) and increased financial burden [7]. Students who undertake an intercalated BSc may develop better strategic learning skills compared to those who do not undertake an additional degree [8], performing better in their examinations in later stages of the medical course than students who do not intercalate [9, 10]. Other advantages offered by intercalation include acquiring a portfolio of new skills, having access to research opportunities which offer an increased likelihood of having papers published and gaining research grants. During their clinical undergraduate training 244 intercalating medical students from Bristol and Sheffield Universities published 54 papers prior to graduation in medicine. Those intercalators with clinical academic supervisors performed significantly better across a number of academic metrics [11].

Medical postgraduate career progression is through Foundation training followed by completion of Core and Higher Specialty programmes. At each stage of the training pathway applications are scored according to the applicants’ experience, achievements and qualifications. Following this, top-scoring applicants are invited to interview where they usually undertake a series of objective assessments [12]. Intercalators, who often have a combination of excellent undergraduate results, an additional qualification in the intercalated degree itself, evidence of research participation and published papers, are well placed to score highly in comparison to those who have not intercalated [9]. Opportunities and experiences at medical school can provide evidence of an interest in and commitment to specialist field of medicine and surgery and enable applicants to stand out at interviews [11, 13].

The Master of Research in Medical Sciences (MRes Med Sci) was developed at The University of Manchester in 2005 as an intercalated Masters degree programme. The MRes Med Sci attempted to bridge the gap between the skills possessed by new medical graduates and those expected for entry to a research degree or an industrial research career. The programme was designed to equip medical students with the knowledge and skills to enable them to pursue a research career in Medicine as clinician scientists. The programme was structured around a split between laboratory-based research and taught elements. Students were matched to research groups for the duration of their intercalated year where they were supervised by Clinical Academics offering translational research projects. This had the advantage of allowing the students to become integrated within their respective research teams and gradually increase the depth and complexity of their research [14]. In addition to expanding the intercalated degree portfolio at The University of Manchester, the MRes Med Sci programme formalised an integrated clinical academic pathway in Manchester; starting in Medical School and progressing through three increasingly senior levels of the Academic Foundation Programme (AFP), Academic Clinical Fellowships (ACFs), Academic Clinical Lectureships (ACLs) and Senior Lectureships during postgraduate training.

Experience in academic medicine during medical school can provide an opportunity to learn valuable generic transferrable skills, provide an opportunity to develop critical thinking and enhance clinical outcomes for patients. However, in studies, medical students rated lifestyle factors and earning potential as more important than the opportunity for research [15] and undoubtedly, the benefits of all types of intercalation need to be better presented to students [7, 11]. Against this background, we hypothesised that research intercalation during medical school would accelerate post-graduate medical career progression. Our aim was to objectively measure the career enhancing impact, quantify the academic benefits and determine the student perception of value of research intercalation at The University of Manchester.

## Methods

### Questionnaire

Data was retrospectively collected, during the spring of 2018, in the form of responses to a questionnaire (Supplementary File 1) sent electronically to alumni of the MRes programme from the years 2005 to 2012. This cohort was selected to permit assessment for a minimum of 5 years post MRes (3–4 years post medical school). The questionnaire was created using the google documents platform. As per the General Data Protection Regulations (GDPR) the MRes programme had retained no personal data pertaining to the MRes alumni and therefore it was not possible to access the contact details of the alumni directly. We therefore asked The University of Manchester Alumni Office to share information about the study with the MRes alumni and invite those interested in participating to contact the study team. We also asked a number of alumni (one from each graduating class), who had remained in touch with the programme director, to advertise the study through their own social media networks and encourage participation. An electronic link to the online survey link was then sent out, followed by weekly reminders over a four-week period.

Informed consent was obtained from all subjects participating in the study. Consent was integral to the self-completion and submission (online) of the questionnaire by the study participants. The questionnaire consisted of three sections: i) demographic information including year of MRes entry; ii) experience of undertaking the MRes degree, including academic outputs, reasons for undertaking the MRes and their opinion of the MRes course; iii) their experience following completion of the MRes intercalated degree: academic sequelae, career progression to date and opinions on whether the MRes had enhanced their career progression. The responses received were anonymised at source.

The study was considered by The University of Manchester Research Ethics Committee and deemed exempt from Research Ethics Approval. An enquiry to the Health Research Authority indicated NHS Research Ethics Committee approval was not required. The study was conducted in accordance with the Declaration of Helsinki.

### Data analysis

The data was exported into Microsoft Excel. Quantitative data was analysed longitudinally to establish a trajectory representing the rate of change over time. Career progression was calculated by comparing the number of years taken for an individual to reach their current position with the minimum number of years in which it was possible to reach that grade [3, 16]. Discrepancies between these variables were investigated to account for career breaks, time spent in non-training positions or appointment to less than full time posts. Completion of higher research degrees (MD or PhD) and professional qualifications (such as MRCP (UK) or MRCS) were recorded. The χ^2^ test was used to compare numbers of publications and presentations made by Manchester MRes students with data previously published from Bristol and Sheffield medical schools [11]. Analysis of the qualitative data was performed by the two members of the study team (MHH and HSY) to ensure consistency in interpretation. Participants with missing data were excluded from the analyses.

## Results

### Demographics

Seventy-seven individuals expressed their interest in participating by contacting the study team for further information, from a total of 142 MRes alumni. Questionnaires were completed by 52 respondents (68% response rate of those who expressed interest). Demographic information was collected from participants to ensure that representative sampling had been achieved and that each MRes year group had multiple respondents. Participants comprised 56% females and 43% males, had a median age of 30.5 years (range: 27–35 years), and had completed the MRes a median of 8 years previously (range: 5–12 years). The majority of respondents had intercalated after Year 4 medicine (94%) and 3.5% of the cohort had been wider participation students.

At the time of participating to the survey all respondents were still in medical practice, 27% (*n*=14), were pursuing clinical academic training or undertaking research fellowships, 73% (*n*=38) were not. The majority of respondents, 58% (*n*=30), were undertaking higher specialty training. A further, 32.5% (*n*=17), were pursuing core medical / surgical training, 2% (*n*=1), foundation training and, 7.5% (*n*=4), were GP principals. The foundation trainee had undertaken their MRes after 3rd year (2012 entry) and had completed their undergraduate studies 2 years prior to the study start date.

### Qualifications and prizes

All participants (*n*=52) had successfully completed their MBChB (medical) and MRes Med Sci qualifications; 2 students had obtained a BSc prior to commencing their MRes. Following graduation, the study cohort successfully completed a further 67 postgraduate qualifications (with an additional 12 qualifications in progress), with each participant achieving a median of 1 postgraduate qualification (range: 0–5). Their postgraduate qualifications included 8 higher degrees (7 PhDs and 1 MD), 4 Masters / Bachelor degrees (3 MSc, 1 MPH, 1 BSc), 41 professional memberships / fellowships (the largest proportion being MRCP (UK), *n*=14 and MRCS, *n*=12) and 12 Diplomas / Certificates (such as Diploma of Child Health, Postgraduate Certificate of Medical Education). In addition, the study cohort achieved a total of 214 under- and post-graduate prizes. Each respondent received at least 1 prize (median 3, range: 1–20). 29% of the cohort achieved 5 or more awards, with 8% achieving 12 accolades or more (Table 1).

**Table 1:** The MRes alumni (*n*=52) achieved 67 postgraduate qualifications with each participant achieving a median of 1 postgraduate qualification (range: 0–5) following graduation from medical school (with MB ChB, MRes Med Sci)

Postgraduate qualifications achieved by the MRes alumni following graduation from medical school (***n***= 52)		
**Higher degree**	**TOTAL**	**8**
	PhD	7
	MD	1
**Degree**	**TOTAL**	**5**
	Masters	4
	BSc	1
**Professional qualifications**	**TOTAL**	**39**
	MRCP (UK)	14
	MRCS	12
	MRCGP	7^a^
	MRCPCH	1
	FRCA	1
	FRCPath	1
	FRCR	1
	FRCOphth	1
	FRCEM	1
	FHEA / AFHEA	3
**Diplomas / Certificates**	**TOTAL**	**12**
	Diplomas	6
	Certificates	6
**In progress**		12

^a^One participant obtained the MRACGP (Membership of the Royal Australian College of General Practitioners)

### Research outputs: papers, presentations and grants

MRes alumni had achieved a variety of publications including peer-reviewed papers and textbook chapters. Responses were received from all alumni (*n*=52) of whom, 84% (*n*=44/52), had published with, 56% (*n*=29/52), of those citing publications as a direct result of their MRes project(s). The MRes cohort published a total of 304 manuscripts giving a median of 3 publications per person (PP, range: 0–53). A total of 55 publications were as a direct result of MRes projects, with one alumnus gaining 6 publications from theirs. Further assessment identified a total of 163 first author publications from 49 alumni, of which 18% (*n*=30/163) of the publications were as a direct result of MRes projects. There were also 18 last author publications from 19% (*n*=10/52) of those individuals participating in the study.

The alumni had presented a large variety of posters and oral presentations at regional, national and international meetings (Table 2). Of the 50 respondents to this survey domain, 96% (*n*=48/50) had made at least 1 presentation. In total the cohort had made 430 presentations, (median 7, range: 0–37), of which 42% (*n*=182/430) were at international meetings. The respondents attributed 17% (*n*=71/430) of their presentations to their MRes projects, of which the majority were to regional/national meetings; 31%, (*n*=22/71) of presentations directly arising from MRes projects were at international conferences.

**Table 2:** The MRes alumni (*n*=50) made 430 presentations, (median 7, range: 0–37), of which 42% (*n*=182) were at international meetings, 17% (*n*=71) of their presentations were attributed to their MRes projects

	Number of presentations (%)	median	mean	range
**Total presentations made by the MRes alumni (*n*=50)**				
Regional	45 (10.5)	0	0.9	0–9
National	203 (47.2)	3	4.06	0–21
Regional + National	248 (57.7)	3.5	4.96	0–22
International	182 (42.3)	2	3.64	0–32
**TOTAL**	**430**	**7**	**8.6**	**0–37**
**Presentations made by the MRes alumni directly attributable to MRes projects (*n*=50)**				
Regional	19 (26.7)	0	0.38	0–3
National	30 (42.3)	0	0.6	0–6
Regional + National	49 (69)	0	0.98	0–7
International	22 (31)	0	0.44	0–4
**TOTAL**	**71**	**0**	**1.42**	**0–9**

A total of 38.5%, (*n*=20/52) of alumni reported research grant funding, with a total of 49 grants. The funding received was disclosed for 43% (*n*=21/49) of the grants, which ranged in value from £250 to £280,000, totalling a cumulative value of £823,000.

Manchester MRes students published significantly more manuscripts (55 papers published from a cohort of 52 students) than those published by intercalators from Bristol and Sheffield medical schools, (Bristol: 12.9%, Sheffield: 38.2% intercalators published [11], *p*< 0.00001, χ^2^ test). In addition, Manchester students made significantly more oral / poster presentations (71 presentations made by 50 students) in comparison to students from Bristol and Sheffield (Bristol: 51.6%, Sheffield: 99% intercalators presented [11], *p*=0.000013, χ^2^ test).

### Career progression and on-going involvement in research

Using the NHS Health Education England career algorithm to define the time required to reach specific career milestones [3, 16], 73%, (*n*=38/52) of the alumni had reached their current positions in the minimum number of years possible. Of those taking longer than the minimum time required for career progression, 27% (*n*=14/52) were attributable to time taken to pursue non-training grade work abroad or to gain locum or clinical fellowship experience prior to starting specialty training. In these individuals a median of 1 year (range: 1–4 years) was added to their career progression.

At the time of survey, 7.7% (*n*=5/52) of our respondents were working overseas. Of these, 40% (*n*=2/5) were in approved non-academic training programmes/clinical fellowships in North America, and 60% (*n*=3/5) were in academic positions (senior lecturer, post-doctoral fellowships) in Australia or the USA. Of the remaining 47 respondents, 91.5% (*n*=43/47) were in a General Medical Council (GMC) recognised training programmes, and 72.1% (*n*=33/43) of these had completed a formal academic component during their post-graduate training, such as AFP, 51.5% (*n*=17/33) and ACF, 33.3% (*n*=11/33). At the time of our survey, 15.15% (*n*=5/33) were undertaking externally funded clinical research training fellowships. Taken together, these data describe post-graduate involvement in research for 73.1% (*n*=38/52) of MRes alumni. However, 19.2% (*n*=10/52) had undertaken no further research during their post graduate training and 7.7% (*n*=4/52) did not provide sufficient employment history for the study team to determine their academic involvement following medical graduation.

### Qualitative views of MRes alumni

When asked if they felt the MRes was a worthwhile experience overall, 98% of alumni stated that it was. When asked to explain what had been gained by MRes intercalation respondents elaborated thus, “a significant increase in my understanding of research … developed skills and understanding not provided by the MBChB programme … publications and presentations” with wider benefit such as “improved work ethic” also. When asked if the MRes had facilitated their career progression, 87% (*n*=45/52) responded that it had, citing factors such as; higher shortlisting scores for foundation and higher speciality training applications, making them more appointable to academic training posts and providing topics for discussion at interview. 12% (*n*=6/52) of respondents stated that the MRes had “maybe” or “to some degree” facilitated their career progression, citing indirect benefits such as learning valuable skills and networking opportunities. Completing the MRes also fostered long lasting support and mentorship for alumni with 54% of respondents acknowledging ongoing contact with their tutors such as providing references, career advice and continuation of projects to doctoral degree programmes after completion of their MRes.

## Discussion

The MRes in Med Sci at The University of Manchester offers medical students an intercalation opportunity which includes the acquisition of a portfolio of transferrable skills, provides foundation training for aspiring clinician scientists and accelerates post-graduate career progression on both academic and non-academic training pathways.

Almost three-quarters of the MRes alumni surveyed had progressed through their post-graduate training in the minimum time, with 87% of study participants indicating that their MRes intercalation had directly facilitated this: “without a doubt the MRes played a pivotal role in my career progression”, the “programme completely changed my career and life. It opened multiple doors for me”. In part this career enhancing effect is due to development of generic professional skills such as time management, team working and self-motivational skills. The clinical research environment clearly added value too, with students given opportunities for networking, career guidance, and mentorship from a faculty of clinical academic supervisors: “I developed skills and understanding not provided by the MBChB programme”. Each MRes student graduated from medical school with (at least) 1 additional degree and following graduation the study cohort successfully completed a further 67 postgraduate qualifications. Other quantifiable outputs included, 55 peer-reviewed publications and 71 national / international presentations, directly attributable to MRes projects. Overall the MRes cohort published a total of 304 manuscripts (median of 3 publications PP, range: 0 to 53) and 430 presentations, (median 7, range: 0–37), of which 42% (*n*=182) were at international meetings. Objective outputs, like publications, offer a significant advantage during post-graduate application processes. For example, applications to higher medical training schemes award 10% of the total shortlisting marks to candidates who are first author, or joint-first author, of two or more PubMed-cited original research publications [17].

Other groups have reported that intercalation per se can benefit a number of academic sequelae [18]. A cross-sectional study examining the benefits of intercalation in two Russell Group institutions also describes significant numbers of publications and oral/poster presentations by students [11]. However, Manchester MRes students published significantly more manuscripts (55 papers published from a cohort of 52 students) and made significantly more oral / poster presentations (71 presentations made by 50 students) in comparison to students from Bristol and Sheffield medical schools. In addition, the majority of our respondents (> 73%) remained research active and pursued further research training following their graduation from the MRes programme and medical school. This is likely to be due to the research focus of the MRes, which may not be provided by other intercalated degree programmes. Furthermore, clinician scientists, who have been shown to augment the wider academic benefits that intercalated degrees provide in other studies [11], supervise all MRes students at The University of Manchester.

Our study did have some limitations. Firstly, due to the retrospective nature of the data collection we were only able to contact 54% of the actual MRes cohort (2005–12). Nevertheless, we achieved a good response to our survey, and we received similar response rates from each year group [19]. However, it is possible that we received responses from those MRes alumni who were most successful during their under-and post-graduate careers, and this may represent a source of self-selection bias in our study. Secondly, we were only able to distinguish between oral and poster presentations for some respondents due to the free-text nature of this domain in our questionnaire. However, we were able to establish whether presentations had been made at international or regional/national level, thus permitting the conference contributions to be appreciated in context. Thirdly, a small number of respondents provided limited employment history, which meant that we could not determine on-going involvement in research, or not, for this group. Finally, our study is limited by the lack of comparison with an appropriate control group. Further prospective evaluation is required, incorporating comparison with peers, on the domains and outcome measures explored in this study.

It is the duty of medical schools to stimulate interest in academic medicine [20]. The MRes intercalated degree equips graduates with the skills required to navigate a clinical academic career but in addition, provides an opportunity to learn valuable generic skills, develop critical thinking and contribute to translational research and improve patient care. These are valuable experiences, and, in this study, we have demonstrated their significant career enhancing potential. However, research intercalation may not suit all medical students. The introduction of a compulsory intercalated degree at an Australian medical school revealed that some students lacked the maturity and personal autonomy required to pursue such a programme [21]. A recent evaluation of the American Medical Scientist Training (integrated MD/PhD) Program identified that a higher proportion of MD/PhD alumni entered faculty positions compared with those graduating medical school who were not on the MD/PhD pathway. However, it remains unclear what outcome measures should be used as measures of success for clinical-academic training and future studies should aim to develop validated criteria for this [22].

It is important to note that the extra years of study also increase the financial burden on students, both in the UK and USA [22]. Following the 2012 increase in the tuition fee cap in England, this may be particularly limiting for some students, especially those from backgrounds with less disposable income. It is important that the potential career-long benefits of intercalation are communicated to students so that informed choice on whether to intercalate or not can be made.

## Conclusion

Research intercalation had a significantly positive impact on postgraduate medical career progression for our cohort and these benefits should be signposted to medical students. Research intercalation specifically enhances entry to clinical academic training and facilitates high-quality research by medical students prior to graduation. This enables early acquisition of transferrable skills may improve career prospects. However, intercalation may be disproportionately accessible to those with disposable income and as such may widen socioeconomic boundaries between graduates. Therefore, widening participation to research intercalation through financial bursaries and scholarships could improve equality among graduates from heterogenous backgrounds.

## Supplementary Information

**Additional file 1.**

## Data Availability

The anonymized datasets used and/or analysed during the current study are available from the corresponding author on reasonable request.

## Abbreviations

NHS: National Health Service
MMC: Modernising Medical Careers
UK: United Kingdom
USA: United States of America
BSc: Bachelor of Science
MRes Med Sci: Master of Research in Medical Sciences
AFP: Academic Foundation Programme
ACF: Academic Clinical Fellowship
ACL: Academic Clinical Lectureship
GDPR: General Data Protection Regulations
GMC: General Medical Council
MD: Doctor of Medicine
PhD: Doctor of Philosophy
MRCP (UK): Membership of the Royal College of Physicians (UK)
MRCS: Membership of the Royal College of Surgeons
χ^2^ test: Chi square test
GP: General Practitioner
MBChB: Bachelor of Medicine, Bachelor of Surgery
MSc: Master of Science
MPH: Master of Public Health
MRCGP: Membership of the Royal College of General Practitioners
MRCPCH: Membership of the Royal College of Paediatrics and Child Health
FRCA: Fellowship of the Royal College of Anaesthetists
FRCPath: Fellowship of the Royal College of Pathologists
FRCR: Fellowship of the Royal College of Radiologists
FRCOphth: Fellowship of the Royal College of Ophthalmologists
FRCEM: Fellowship of the Royal College of Emergency Medicine
FHEA: Fellowship of the Higher Education Academy
AFHEA: Associate Fellowship of the Higher Education Academy
PP: Per person

## References

[1] Philip AB, Prasad SJ, Patel A (2015). Should an intercalated degree be compulsory for undergraduate medical students?. Med Educ Online.

[2] Department of Health (2000). The NHS plan. A plan for investment. A plan for reform, CM4818-I.

[3] Department of Health (2003). Modernising medical careers: the response of the four UK Health Ministers to the consultation on unfinished business: proposals for reform of the senior house officer grade.

[4] Griggs I (2007). Babies ‘will be put at risk’ as experienced doctors are replaced by trainees. The Independent.

[5] Walport M (2005). UK clinical research collaboration. Medically- and dentally-qualified academic staff: recommendations for training the researchers and educators of the future.

[6] Irwin GW, Spence RAJ, McAuley DF, Kennedy RD, Gardiner KR, Elborn JS (2014). Academic medicine - revolution, evolution or extinction?. Ulster Med J.

[7] Nicholson JA, Cleland J, Lemon J, Galley HF (2010). Why medical students choose not to carry out an intercalated BSc: a questionnaire study. BMC Med Educ.

[8] McManus IC, Richards P, Winder BC (1999). Intercalated degrees, learning styles and career preferences: prospective longitudinal study of UK medical students. BMJ.

[9] Cleland JA, Milne A, Sinclair H, Lee AJ (2009). An intercalated BSc degree is associated with higher marks in subsequent medical school examinations. BMC Med Educ.

[10] Mahesan N, Crichton S, Sewell H, Howell S (2011). The effect of an intercalated BSc on subsequent academic performance. BMC Med Educ.

[11] Stubbs TA, Lightman EG, Mathieson P (2013). Is it intelligent to intercalate? A two Centre cross-sectional study exploring the value of intercalated degrees, and the possible effects of the recent tuition fee rise in England. BMJ Open.

[12] Medical training pathway (2020). British Medical Association.

[13] Mabvuure NT (2012). Twelve tips for introducing students to research and publishing: a medical student’s perspective. Med Teach.

[14] Faculty of Medical and Human Sciences, Manchester Medical School (2011). MRes Med Sci 2011–12 Handbook.

[15] Park SJ, McGhee CN, Sherwin T (2010). Medical students’ attitudes towards research and a career in research: an Auckland, New Zealand study. N Z Med J.

[16] Health Careers. Explore Roles. NHS Health Education England. Available from: https://www.healthcareers.nhs.uk/explore-roles Accessed 21 Apr 2020.

[17] NHS Physician ST3 Recruitment. Application scoring. In: Guidance on the scoring process and ‘evidence’ page of the ST3 application form: Health Education England; 2020. Available from: http://www.st3recruitment.org.uk/recruitment-process/applying/application-scoring Accessed 21 Apr 2020.

[18] Agha R, Fowler A, Whitehurst K, Rajmohan S, Gundogan B, Koshy K (2017). Why apply for an intercalated research degree?. Int J Surg Oncol.

[19] Fincham JE (2008). Response rates and responsiveness for surveys, standards, and the journal. Am J Pharm Educ.

[20] Straus SE, Straus C, Tzanetos K (2006). International campaign to revitalise academic medicine. Career choice in academic medicine: systematic review. J Gen Intern Med.

[21] Collins JP, Farish S, McCalman JS, McColl GJ (2010). A mandatory intercalated degree programme: revitalising and enhancing academic and evidence-based medicine. Med Teach.

[22] Brass LF, Akabas MH (2019). The national MD-PhD program outcomes study: relationships between medical specialty, training duration, research effort, and career paths. JCI Insight.

