# Supplementary material for: The impact of research intercalation during medical school on post-graduate career progression
Source: BMC Med Educ. 2021 Jan 8;21:39. doi: 10.1186/s12909-020-02478-7 (PMC7792318; doi:10.1186/s12909-020-02478-7)
Supplement: Supplementary file 1 — Additional file 1. [file 12909_2020_2478_MOESM1_ESM.pdf]

The Impact of Research Intercalation during Medical School on Post-Graduate Career Progression.

Antony K Sorial[1*], Morgan Harrison-Holland[2*], Helen S Young[1]

[1]Biosciences Institute, Newcastle University, Newcastle upon Tyne, UK

[2]Department of Dermatology, The University of Manchester, Oxford Road, Manchester, UK

**AKS and MHH are joint first authors*

Corresponding author

Helen Young

Department of Dermatology,

The University of Manchester, Salford Royal Hospital NHS Foundation Trust,

Stott Lane, Manchester, UK

Telephone: 0161 206 0523   Fax: 0161 206 1029

Email: helen.s.young@manchester.ac.uk

ORCID: 0000-0003-1538-445X

MRes Feedback Form

* Required

1. Please state your gender *

   Mark only one oval.

   - Male
   - Female
   - Do not wish to disclose

2. Please state your current position *

   ____

3. Have you always worked full-time? If no, how many years have you worked parttime?

   ____

4. Please select your year of entry to the MRes *

   Mark only one oval.

   - 2005
   - 2006
   - 2007
   - 2008
   - 2009
   - 2010
   - 2011
   - 2012
   - 2013
   - 2014

5. Please state your current age *

6. Please state your qualifications at the time of undertaking MRes *

Select all that apply

Check all that apply.

- [ ] None (undertook MRes after 3rd year of MB ChB)
- [ ] None (undertook MRes after 4th year of MB ChB)
- [ ] BSc
- [ ] MSc
- [ ] MB ChB
- [ ] Academic Clinical Fellow

Other: [ ]

7. Please state your career objectives prior to MRes *

8. Did you undertake one or two projects during your MRes? *

Mark only one oval.

- ( ) One
- ( ) Two

9. Please select all your undergraduate and postgraduate qualifications to date (in addition to your MRes) *

Select all that apply

Check all that apply.

- [ ] MB ChB
- [ ] BSc
- [ ] MSc
- [ ] MD
- [ ] PhD
- [ ] MRCP Part 1
- [ ] MRCP Part 2 (written)
- [ ] MRCP PACES
- [ ] MRCS Part A
- [ ] MRCS Part B OSCE

Other: [ ] ____________________

10. Please list all prizes/merits/distinctions/honours awarded during and after medical school

____

____

____

11. Please list all your publications to date (please include full citation details).
Please add an asterisk '*' to those which are directly related to your MRes

____

____

____

12. Please list all your presentations to date (please specify whether they are local, national or international).Please add an asterisk '*' to those which are directly related to your MRes

13. Please list any grants awarded

14. Please state your career progression since leaving MRes (please specify the post, specialty, whether clinical/academic work, and type of contract) *

15. What were your reasons for choosing to enrol into the programme? *

16. What did you gain from the programme? *

17. Has the programme facilitated your career progression? If yes, please provide more details. *

18. Describe any difficulties you had in combining clinical and academic training whilst at medical school or after graduating.

19. Overall, was the MRes Programme a worthwhile experience? *

Mark only one oval.

- Yes
- No

20. Were you a wider participation student at the University of Manchester? *

Mark only one oval.

- Yes
- No

21. Were you a Wider Participation Fellow at the University of Manchester during your MRes? *

Mark only one oval.

- Yes
- No

22. As an MRes alumnus, would any of the following be of interest to you?
Select all that apply

Check all that apply.

- [ ] Networking events
- [ ] Online network (hosted on University of Manchester website)
- [ ] Facebook group
- [ ] LinkedIn group

Other: [ ] ______________________

23. Had you spent time with your MRes project supervisor/group prior to your MRes? Please describe *

____

____

____

24. Have you received any ongoing support from your MRes supervisors (e.g. references, further laboratory experience) following your MRes year. Please describe *

25. If you are happy to be contacted by email, please provide below
Optional

26. If you are happy to, please write down your name so we can keep track of who has answered the survey (optional).

This content is neither created nor endorsed by Google.

Google Forms
